# Spherical Composite Powder by Coupling Polymethyl Methacrylate and Boron Nitride via Spray Drying for Cosmetic Application

**DOI:** 10.3390/ma12050706

**Published:** 2019-02-28

**Authors:** Cherng-Yuh Su, Jia-Chang Wang, Chih-Yuan Chen, Kent Chu, Chung-Kwei Lin

**Affiliations:** 1 Department of Mechanical Engineering, National Taipei University of Technology, Taipei 106, Taiwan; cysu@ntut.edu.tw (C.-Y.S.); jcw@ntut.edu.tw (J.-C.W.); nb0693@gmail.com (C.-Y.C.); 2 Additive Manufacturing Center for Mass Customization Production, National Taipei University of Technology, Taipei 106, Taiwan; 3 Research Center of Digital Oral Science and Technology, College of Oral Medicine, Taipei Medical University, Taipei 110, Taiwan; 4 HeBN Co., Ltd., Taichung 411, Taiwan; kent@nntbn.com.tw; 5 School of Dental Technology, College of Oral Medicine, Taipei Medical University, Taipei 110, Taiwan

**Keywords:** blemish balm cream, boron nitride, PMMA, optical properties, sun protection factor (SPF), cosmetics, thermal conductivity

## Abstract

In the present study, spherical composite powder was successfully prepared via spray drying process using polymethyl methacrylate (PMMA) and hexagonal boron nitride (h-BN) powders. The pristine and as-prepared composite powders were examined using scanning electron microscopy, a particle size analyzer, oil absorption, and specific surface area analyses. These powders were then mixed with linseed oil to prepare samples for UV-Visible-Near Infrared spectroscopy investigation to determine their light absorption ability. Blank and powder-added blemish balm creams were examined using a sun protection factor tester and a thermal conductivity tester. In addition, transmittances of these creams were also evaluated. The experimental results show that spray-dried spherical composite powder exhibited good oil absorption ability. The blemish balm cream with 10 wt.% spray-dried composite powder not only exhibited superior sunscreen protection ability, but also good thermal conductivity.

## 1. Introduction

Solar radiation emitting from the sun covers a wide range of electromagnetic wave spectra, including those in the ultraviolet (200–400 nm), visible (400–760 nm), and infrared (760–4000 nm) regions [1]. Infrared and ultraviolet radiation are detrimental to human skin in different ways. Radiation types within the near-infrared range (abbreviated as NIR or IRA, 760–1440 nm) that possess longer wavelengths can penetrate deeper into skin, allowing it to reach the subcutis, and induce photoaging of the skin [2,3]. Ultraviolet radiation can be divided into UVC (200–290 nm), UVB (290–320 nm), and UVA (320–400 nm). Although UVC has the highest energy, most UVC light is blocked by the ozone layer [4]. UVB and UVA light, however, can penetrate human skin and cause damage. UVB is mainly absorbed in the epidermis, which can induce redness and damage DNA [5]. UVA possessing relatively longer wavelengths can reach the epidermis and dermis, which can cause slight sunburn, as well as degenerate collagen and elastin within the dermis layer. A consequence of this is a decrease in skin elasticity that results in skin wrinkles.

Sunscreen and Blemish Balm cream are cosmetics commonly used to protect skin from solar radiation. Organic and/or inorganic compounds are added to these cosmetics to serve as sunlight filters [6,7]. The organic compounds usually possess aromatic bonds. When these bonds are exposed to solar radiation, they can undergo conformational changes, emit radiation with lower energy, or release the absorbed energy as heat [8]. The reaction mechanism of organic compounds is due to chemical molecular changes that occur upon receiving solar radiation. Avobenzone [9], oxybenzone [10], and others [11] are examples of typical organic sunscreens. Inorganic sunscreens, on the other hand, use physical sunscreen agents that can absorb, reflect, and scatter solar radiation. Titanium dioxide and zinc oxide are two of the most frequently used inorganic compounds in cosmetics and are approved by the *Food and Drug Administration* (FDA) [12]. Their usage as micron-sized particles has shifted to nanoscaled ones [12,13,14]. Modifications have also been attempted to further improve their performance [15,16,17]. In addition to TiO_2_ and ZnO, boron nitride has attracted increasing R&D attention [18,19,20,21,22,23]. The Cosmetic Ingredient Review Expert Panel evaluated the safety issues of boron nitride and concluded that it is safe in cosmetics in the present practices of use and concentration [23,24]. Hexagonal boron nitride (h-BN), which is similar to graphite, exhibits a layered structure and possesses unique characteristics, such as a lubricating effect, excellent thermal stability, and oxidation resistance. Incorporation of h-BN with other cosmetic ingredients has been attempted, expanding its application in cosmetics [25,26,27,28].

Since different organic and/or inorganic compounds offer better protection within specific solar spectrum ranges, a combination of various ingredients is commonly used in commercial cosmetic formulations to obtain desired levels of sun protection. In either case, compounds in the form of spherical powder, when compared to irregular-shaped or flake-like powder, exhibit more uniform reflectivity, superior dispersion, improved viscosity and better flow ability. Thus, coupling organic and/or inorganic materials and making them spherical is an important issue in cosmetic applications [18,25,26,29,30]. For instance, polymethyl methacrylate (PMMA), which is approved by the FDA for its use in certain medical devices, is a polymer used in the cosmetic industry as film formers and agents to improve viscosity [31]. More importantly, PMMA alone or combined with other materials can be easily made into spherical powders and used as cosmetics [29].

Spray drying process is an efficient method to produce spherical powders from a liquid or slurry that is atomized and quickly dried by a hot air environment. In this study, spray drying process was used to couple boron nitride and PMMA into a spherical composite powder. During the spray drying process, h-BN can be randomly embedded into molten or semi-molten PMMA to form spherical particles. After performing particle size measurement and structural investigations, the pristine and as-prepared composite powders were mixed with linseed oil or blemish balm creams to evaluate their cosmetic performance.

## 2. Experimental

Commercially available PMMA (GW-T series, GuideWin Special Chemical Co. Ltd., Taoyaun, Taiwan) and BN powder (CNA04, National Nitride Technologies Co., Ltd., Taichung, Taiwan) were used as the starting materials for the spray drying process. After testing various weight ratios and processing parameters, PMMA and h-BN (weight ratio of PMMA to h-BN = 1:3) powder were separately added into deionized (DI) water (weight ratio of powder to DI water ratio = 1:4) to prepare powder-containing solutions. These solutions were then mixed together, magnetically stirred, and ball milled to obtain a homogenous PMMA and h-BN dispersed solution for the spray drying process. A CNK SDDNO-3 spray drying system (IDTA Machinery Co., Ltd., Taipei, Taiwan) was used to spray the PMMA/h-BN composite powder with the following process parameters: in air = 200–220 °C, out air = 80–120 °C, disc rotation speed = 30,000 rpm, and a feed rate of ~1.5 kg/h. The spray dried composite powder was examined using a field emission scanning electron microscope (JSM-6500F, JEOL Co., Ltd., Tokyo, Japan) to determine its powder morphology and cross section. The particle size distribution was obtained using a dynamic light scattering particle size analyzer (SALD-201V, SHIMADZU Co., Ltd., Kyoto, Japan). The specific surface areas of powder were examined using a surface area analyzer (ASAP2010, Micromeritics Instrument Corp., Norcross, GA, USA) with nitrogen gas adsorption using the BET method, while the oil absorption ability of the powder was investigated according to JIS-K5101/ISO787-5 standard [32,33]. The powder-added oil samples were prepared by mixing h-BN, PMMA, and h-BN/PMMA composite powder (coded as DP25B75) with linseed oil and placing the mixtures into a quartz plate with a size of 10 × 10 × 1 mm^3^ for light absorption evaluation ranging from 220–1440 nm (i.e., UVC-NIR range) using a UV-VIS spectrophotometer (UV-3600 UV-VIS-NIR Spectrophotometer, SHIMADZU Co., Ltd., Kyoto, Japan).

Usually limited amounts (say less than 10 wt.%) of individual inorganic physical sunscreen agents are added to make sun protection creams. In order to investigate their cosmetic properties in practical use, 3 and 10 wt.% pristine h-BN and PMMA/h-BN composite powders were added to Blemish Balm cream (i.e., BB cream, obtained from Kalin Enterprise Co., Ltd., Taipei, Taiwan), separately. Table 1 summarizes the information of ingredients for the blank and powder-added BB creams. Firstly, the oil-phase ingredients were placed in a stainless steel beaker and mixed by a mixer for 5 min. Subsequently inorganic additives were added into the beaker and mixed further for 20 min. Finally, water-phase ingredients were slowly poured into the beaker and mixed for another 5 min to obtain the powder-added BB creams. Sun protection factor (SPF), light transmittance within the UVC-NIR region, and thermal conductivity of powder-containing BB cream were examined. The SPF value within a wavelength length range of 290 to 400 nm was measured using a sun protection factor tester (SPF-290S, Optometrics Co., Ltd., Littleton, MA, USA), for which the samples were coated on a 3M transparent film with an area of 2 mg/cm^2^. The SPF values were obtained from six different locations on the film to determine the SPF value of each sample [34]. Transmittance within the UVC-NIR region was determined using a UV-3600 UV-VIS-NIR Spectrophotometer. In addition, the thermal conductivity was measured according to the ASTM D-5470-01 standard [35] using a guarded heater with a reference calorimeter [35,36].

## 3. Results and Discussion

**Figure 1:**
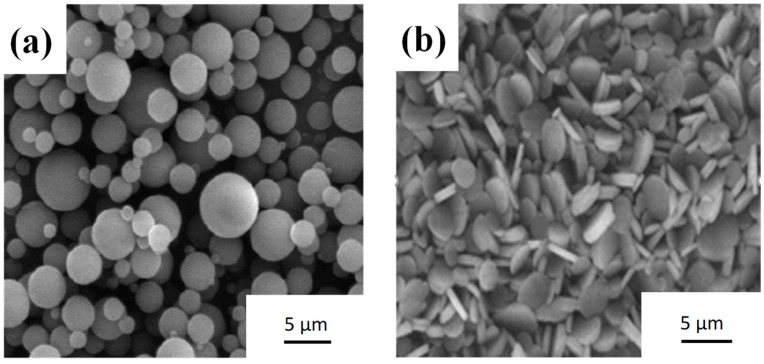
SEM images of (**a**) PMMA and (**b**) h-BN powder.

**Figure 2:**
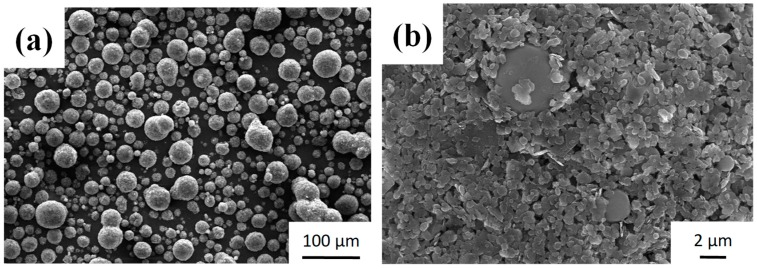
(**a**) Powder morphology and (**b**) cross-sectional view of DP25B75 powder.

**Figure 3:**
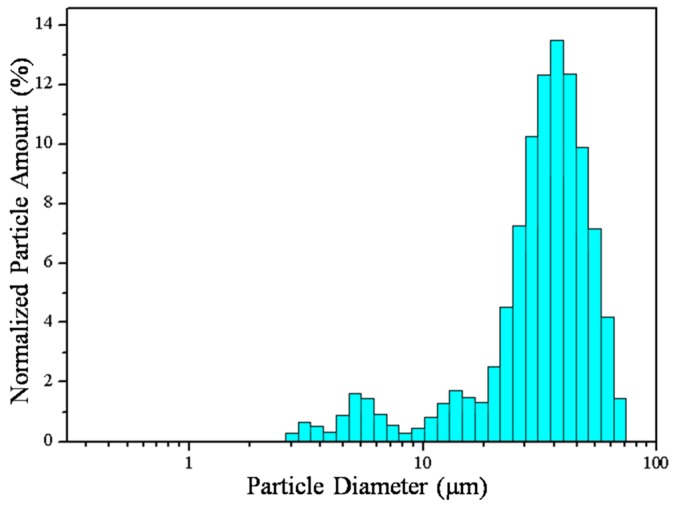
Particle size distribution of DP25B75 powder.

Powder morphology may affect the preparation of cosmetics. Figure 1 shows the SEM images of the starting PMMA and h-BN powder. PMMA is spherical with an average size of ~4 μm, as shown in Figure 1a, whereas boron nitride powder, shown in Figure 1b, is plate-like with an average size of ~2 μm. Figure 2 shows the spray dried DP25B75 composite powder. It can be noted from Figure 2a that sphere-like particles formed after the spray drying process. Figure 2b shows the cross-sectional view of DP25B75 powder, in which the plate-like h-BN powder was homogeneously distributed within a melted or semi-melted PMMA matrix. Figure 3 shows the particle size distribution of DP25B75 composite powder, for which a negatively skewed distribution or a multi-model distribution can be noted. The largest peak in Figure 3 (i.e., the mode) is 41.5 μm and the median size of DP25B75 powder is 30.5 μm, i.e., indicating that 50% of the particles are smaller than 30.5 μm and the other 50% are larger than 30.5 μm. The median size of DP25B75 is smaller than that of the TiO_2_/mica-BN powder (~60 μm) prepared through a similar process [26].

**Figure 4:**
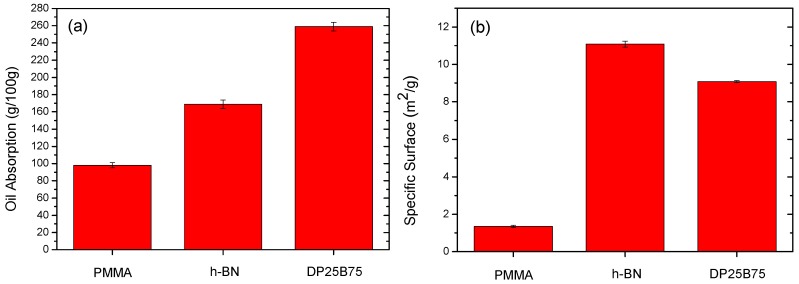
(**a**) Oil absorption and (**b**) specific surface area of PMMA, h-BN, and DP25B75 powder.

For practical cosmetic application, the oil absorption property is important and Figure 4a shows the oil absorptions of pristine PMMA, h-BN, and DP25B75 composite powders. Spherical PMMA with an average particle size of 4 μm exhibited an oil absorption of 98 g/100 g. Plate-like h-BN with a smaller particle size (2 μm) exhibited a better oil absorption property of 169 g/100 g, whereas DP25B75 exhibited a superior oil absorption ability of 259 g/100 g. It should be noted that spray-dried DP25B75 composite powder has a median size of 30.5 μm, which is significantly larger than that of pristine PMMA and h-BN powder. In order to clarify the oil absorption performance of these powders, their specific surface areas were shown in Figure 4b. The specific surface area of spherical PMMA and plate-like BN is 1.35 and 11.08 m^2^/g, respectively. DP25B75, however, exhibited a surface area of 9.09 m^2^/g, which is between that of PMMA and h-BN. As shown in Figure 2b, the cross-section of DP25B75 revealed that the spray-dried powder possessed numerous pores. Though the specific surface area (measured by N_2_ gas absorption) is smaller than that of h-BN powder, the oil absorption of DP25B75 is apparently larger than that of BN powder. This can be attributed to the numerous pores formed within the composite powder during the spray drying process.

**Figure 5:**
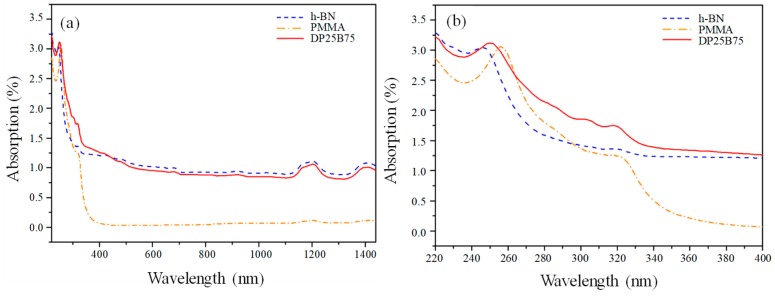
(**a**) UVC–near infrared (220–1440 nm) and (**b**) UVC–UVA (220–400 nm) spectra for powder-added oil samples prepared by adding PMMA, h-BN, and DP25B75 powder.

Figure 5a shows the light absorption properties of powder-added oil samples prepared by adding PMMA, h-BN, and DP25B75 powder into linseed oil. It can be noted that, within the UVA-NIR range (320–1440 nm), the powder-added oil sample with PMMA exhibited a significantly smaller light absorption ability compared to those prepared by adding h-BN or DP25B75. The light absorption abilities of these materials, however, were very close at relatively short wavelength regions within 220–320 nm (i.e., UVC-UVB ranges). Figure 5b shows the details of the light absorption within the UVC-UVA regions. Each of them exhibited an absorption peak at the UVC region (100–280 nm). The absorption peaks are 247, 249, and 256 nm for h-BN, DP25B75, and PMMA, respectively. It is interesting to note that the absorption peak of DP25B75 (i.e., 249 nm) equals the weighing of those of h-BN and PMMA. Even at higher wavelengths up to 400 nm, the spray-dried DP25B75 composite powder exhibited a better light absorption performance than the starting materials.

**Figure 6:**
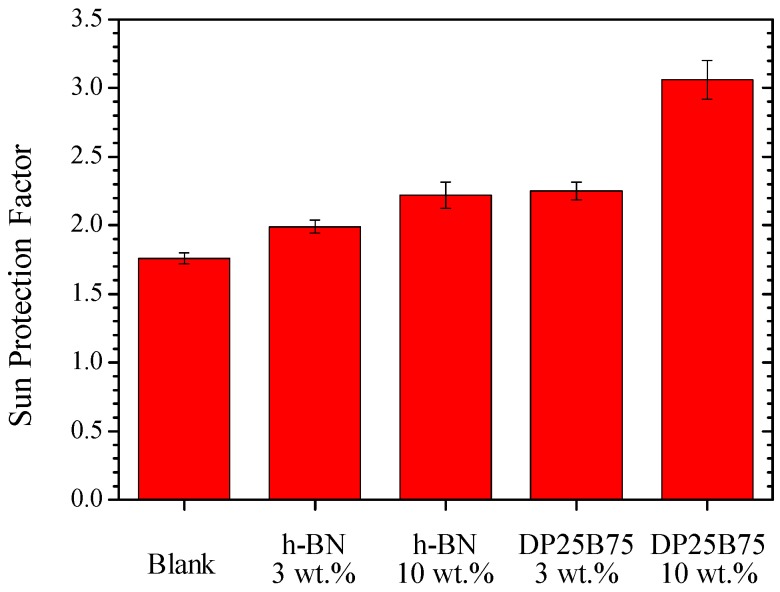
Sun protection factor (SPF) of BB creams with or without h-BN and DP25B75 powder.

To simulate practical cosmetic applications, 3 and 10 wt.% h-BN or DP25B75 powder was added to Blemish Balm cream (i.e., BB cream). The sun protection factor, transmittance within UVC-NIR regions, and thermal conductivity of pristine and powder-added BB creams were investigated. Figure 6 shows the sun protection factors of pristine and powder-added BB creams. Blank BB cream exhibits a SPF of 1.76 that apparently increases after powder additions. The SPF is 1.99 and 2.22 with 3 and 10 wt.% h-BN addition, respectively. This is further improved when using DP25B75 composite powder. With 3 wt.% DP25B75 addition, the SPF is 2.25, which is higher than that of 10 wt.% h-BN. With 10 wt.% DP25B75, the SPF is 3.06, which is ~74% higher than that of pristine BB cream and ~38% higher than its h-BN counterpart. The SPF increases with increasing amounts of DP25B75 powder additions. Desired SPF may be achieved by adding more amount of DP25B75 powder or chemical organic agents. The larger the SPF is, the better the light absorption in the ultraviolet light region [37]. This indicates that BB cream with spray-dried DP25B75 composite powder exhibited superior sunscreen protection compared to its pristine h-BN counterpart.

**Figure 7:**
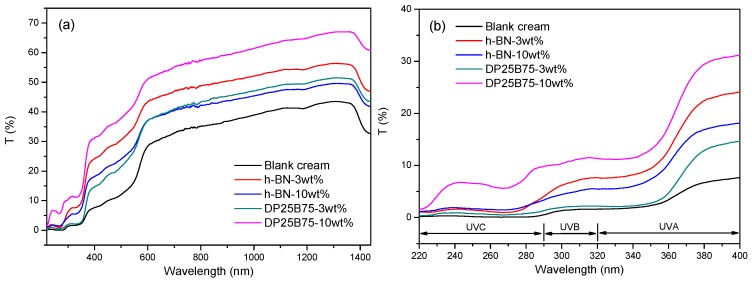
(**a**) UVC-near infrared (220–1440 nm) and (**b**) UVC-UVA (220–400 nm) spectra of BB creams with or without h-BN and DP25B75 powder.

Figure 7a shows the transmittance spectra of light within the UVC-NIR regions. As a general trend, transmittance of light increases with increasing wavelength for all the BB creams with or without powder additions. It can also be noted that the transmittance of BB cream decreases after powder addition. Small amounts of spherical DP25B75 powder (3 wt.%) decreases light transmittance but the effect is poorer than of its plate-like h-BN counterpart. The differences between adding 3 or 10 wt.% h-BN is not significant. The best light occlusion can be observed with 10 wt.% DP25B75 composite powder. The transmittance spectra for these BB creams is shown in Figure 7b and shows a trend similar to the one discussed above. This suggests that a suitable amount of spherical composite powder, such as 10 wt.%, can exhibit a superior light occlusion performance.

**Figure 8:**
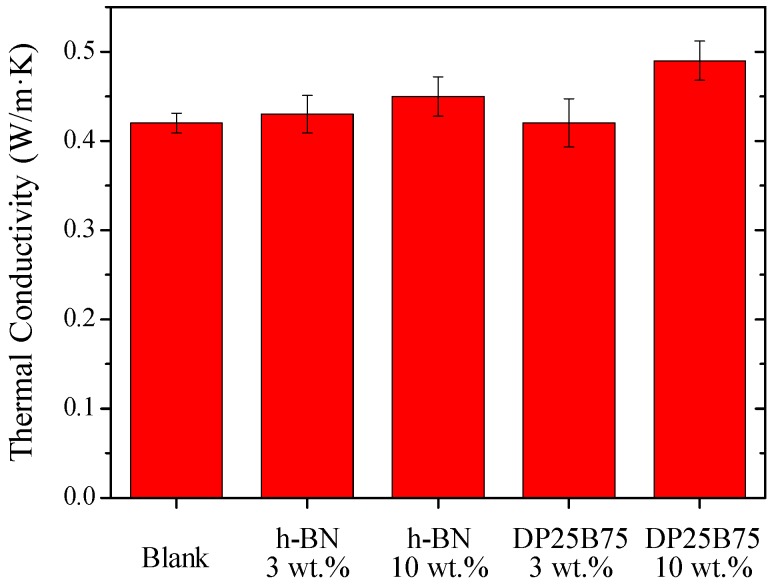
Thermal conductivity of BB creams with or without h-BN and DP25B75 powder.

Thermal conductivity indicates the cooling effect on the skin after usage [38] and Figure 8 shows the thermal conductivity of blank and powder-added BB creams. The thermal conductivity is 0.42 W·m^−1^·K^−1^ for blank BB cream and exhibits no obvious improvement with only 3 wt.% of h-BN (0.42 W·m^−1^·K^−1^) or DP25B75 (0.43 W·m^−1^·K^−1^) addition. Significant improvement of thermal conductivity can be noticed with the addition of 10 wt.% h-BN and DP25B75. The thermal conductivity is 0.45 and 0.49 W·m^−1^·K^−1^ for h-BN and DP25B75, which corresponds to an increase of 7 and 17%, respectively.

## 4. Conclusions

Using PMMA and h-BN as starting materials, DP25B75 composite powder was successfully prepared via spray drying process. Spray-dried DP25B75 was spherical and exhibited a median size of 30.5 μm, forming structures in which h-BN powder was embedded within a melted or semi-melted PMMA matrix. The oil absorption ability of DP25B75 was 259 g/100 g, which is significantly higher than that of PMMA (98 g/100 g) or h-BN (169 g/100 g) and can be attributed to the numerous pores within the spray-dried composite powder. The light absorption abilities of powder-added oil samples were very close, falling within UVC-UVB ranges (i.e., 220–320 nm) and exhibiting absorption peaks at 247, 249, and 256 nm for h-BN, DP25B75, and PMMA, respectively. PMMA-added oil samples generally exhibited a light absorption ability less than that of h-BN or DP25B75. For practical BB cream application, both the sun protection factor and thermal conductivity were improved with powder additions. For Blemish Balm cream with 10 wt.% DP25B75 composite powder, the SPF reached 3.06, which corresponds to a 74% and 38% improvement compared to that of pristine BB cream and the h-BN counterpart respectively. The thermal conductivity was 0.49 W·m^−1^·K^−1^, which was better than that of the blank BB cream (0.42 W·m^−1^·K^−1^) and the h-BN cream (0.45 W·m^−1^·K^−1^). Additionally, 10 wt.% DP25B75-added BB cream exhibited the best light occlusion performance within the UVC-NIR regions.

## Figures and Tables

**Table 1:** Ingredients of blank and powder-added BB creams.

Classification	Ingredient	INCI *	Blank (wt.%)	Powder 3 (wt.%)	Powder 10 (wt.%)
Oil-phase ingredients	TSF-405	Cyclomethicone	16		
	2-EHP	Octyl Palmitate	5		
	Salacos 913	Isotridecyl Isononanoate	5		
	Amiter LG-1600	Dihexyldecyl Lauroyl Glutamate	1		
	Emalex DISG-2EX	Polyglyceryl-2 Diisostearate	2		
	Emalex SS-5051	PEG-10 Dimethicone/Dimethicone	4		
	Tocopherol 80	Tocopherol	0.1		
	Phenyloxyethanol	Phenyloxyethanol	0.3		
Inorganic additives	S-BEN(W)	Quaternium-18 Bentonite	2		
	W704	Titanium Dioxide/Talc/Mica/Lauroyl Lysine/Iron Oxide	8		
	Present work h-BN or DP25B75	Boron Nitride or Boron Nitride/Polymethyl Methacrylate	0	3	10
Water-phase ingredients	1,3-BG	Butylene Glycol	3		
	Sodium Lactate (60%)	Sodium Lactate	2		
	EDTA-2Na	EDTA-2Na	0.05		
	Deionized water	Water	51.55	48.55	41.55

* INCI: International Nomenclature of Cosmetic Ingredients.
